# Supplementary material for: Research on technology innovation path of Intelligent Manufacturing enterprises—Based on qualitative comparative analysis of fuzzy sets under TOE framework
Source: PLoS One. 2024 Oct 24;19(10):e0309784. doi: 10.1371/journal.pone.0309784 (PMC11500965; doi:10.1371/journal.pone.0309784)
Supplement: S1 File — (DOCX) [file pone.0309784.s001.docx]

**Raw data**

| **Code** | **companies** | **R&D** | **Digital Transformation** | **human capital** | **ROA** | **government support** | **competitive position** | **Number of intellectual property rights** |
| --- | --- | --- | --- | --- | --- | --- | --- | --- |
| 000100 | TCL TECH | 0.051837 | 1.6094 | 26.79% | 0.004966883 | 0.007942124 | 0.054854 | 2313 |
| 000157 | ZOOMLION | 0.06023 | 3.4012 | 45.38% | 0.019300523 | 0.017187845 | 0.117193 | 349 |
| 000338 | Weichai Power | 0.044138 | 3.8501 | 38.58% | 0.01935086 | 0.004119067 | 0.070756 | 831 |
| 000425 | XuGong | 0.043513 | 2.9444 | 48.55% | 0.024528951 | 0.004017799 | 0.105651 | 31 |
| 000988 | HUAGONG TECH | 0.047095 | 2.8904 | 56.46% | 0.05303291 | 0.007247465 | 0.105593 | 108 |
| 001339 | JWIPC TECHNOLOGY | 0.055008 | 4.0604 | 25.29% | 0.03949569 | 0.001730616 | 0.110432 | 7 |
| 002008 | Han's Laser | 0.107474 | 2.7081 | 41.7% | 0.04016642 | 0.009322071 | 0.186052 | 133 |
| 002415 | Hikvision | 0.11801 | 4.7791 | 68.03% | 0.11370122 | 0.008732736 | 0.273602 | 605 |
| 002594 | BYD | 0.04399 | 2.7081 | 13.44% | 0.035866604 | 0.004033598 | 0.11125 | 112 |
| 002747 | ESTUN | 0.079257 | 2.7081 | 44.74% | 0.022162869 | 0.010691745 | 0.161797 | 15 |
| 300161 | Huazhong | 0.146317 | 3.9120 | 34.62% | 0.00156546 | 0.07613188 | 0.147417 | 38 |
| 300433 | Lens Technology | 0.045076 | 3.7136 | 3.88% | 0.032163445 | 0.01065768 | 0.118544 | 36 |
| 300450 | Wuxi | 0.096745 | 2.1972 | 32.13% | 0.070429175 | 0.002765752 | 0.29638 | 92 |
| 300678 | Information Technology Of Chinese | 0.057704 | 5.0106 | 91.83% | 0.041987503 | 0.026104672 | 0.145942 | 12 |
| 300750 | CATL | 0.047202 | 1.0986 | 20.86% | 0.055673538 | 0.008223004 | 0.147496 | 415 |
| 600031 | SanyHeavy | 0.086517 | 3.5264 | 50.64% | 0.027744613 | 0.011004223 | 0.128444 | 683 |
| 600089 | TBEA | 0.013318 | 2.3979 | 52.63% | 0.134165273 | 0.00441637 | 0.3295 | 47 |
| 600150 | CSSC Holdings | 0.051584 | 2.0794 | 53.32% | 0.005371003 | 0.01312518 | 0.010262 | 1247 |
| 600582 | Tiandi Science | 0.064298 | 2.4849 | 43.1% | 0.058345949 | 0.008409573 | 0.186023 | 630 |
| 600690 | Haier Smart Home | 0.039009 | 3.7842 | 33.2% | 0.062466252 | 0.003147367 | 0.110282 | 65 |
| 600835 | Shanghai Mechanical&Electrical Industry | 0.033478 | 1.3863 | 38.1% | 0.040897249 | 0.002457697 | 0.092466 | 249 |
| 601766 | CRRC | 0.058894 | 1.9459 | 43.63% | 0.032460692 | 0.008846067 | 0.117463 | 1223 |
| 601877 | Zhejiang Chint Electrics | 0.024719 | 3.9703 | 24.19% | 0.045237248 | 0.003887668 | 0.150094 | 73 |
| 603496 | EmbedWay Technologies | 0.190104 | 4.9836 | 73.74% | 0.044031719 | 0.004144412 | 0.289325 | 8 |
| 688128 | China National Electric Apparatus | 0.075768 | 3.6889 | 63.94% | 0.057304283 | 0.017292367 | 0.158337 | 25 |
| 688777 | SUPCON Tech | 0.104512 | 4.8283 | 73.87% | 0.061803241 | 0.008072088 | 0.205755 | 147 |
