# Supplementary material for: Research on technology innovation path of Intelligent Manufacturing enterprises—Based on qualitative comparative analysis of fuzzy sets under TOE framework
Source: PLoS One. 2024 Oct 24;19(10):e0309784. doi: 10.1371/journal.pone.0309784 (PMC11500965; doi:10.1371/journal.pone.0309784)
Supplement: Attachment — Submitted filename: 1.Response to Reviewers.docx [file pone.0309784.s002.docx]

Dear Editor and Reviewers,

We are grateful for the time and effort you have invested in reviewing our manuscript. We have carefully considered all the comments and suggestions provided and have made revisions to address the concerns raised. Below, we provide a detailed point-by-point response to each comment.

Reviewer #1:

Comment : The “Introduction” part should be enriched. The “Literature Review” part should include more latest literatures. The “Methods” part should be elaborated more. The “Results” part is well-written. The study should include “Policy Implications”.

Response:

We appreciate this suggestion.

We have added and adjusted the Introduction part on page 3, 4

We have added and adjusted the Literature Review part on page 7, 8,9, 11

We have added and adjusted the Methods part on page 18,19, 20

We have added and adjusted the Policy Implications part on page 38,39, 40

Reviewer #2:

Comment 1:What are the techniques other than Fuzzy Set Qualitative Comparative Analysis (fsQCA) technique that can be used in the current study.

Comment 2:What makes the fsQCA more appropriate in comparison to the those techniques?

Comment 3:Can the authors provide any reference to the statement in section 3.3 "after the original consistency threshold was adjusted, which indicates that the results of this paper are robust".

Response:

We appreciate this suggestion.

We have added additional content to the Comment 1 on page 19, 20

We have added additional content to the Comment 2 on page 19, 20

We have added additional content to the Comment 3 on page 35, 36

Reviewer #3:

Comment 1:Sentences written are quite big in length (for eg: see the first sentence of the abstract:- quite lengthy.) These should be restructured into smaller readable parts.

Comment 2:Use of proper punctuation is required to form the sentences. Without doing this it is impossible to read them.

Comment 3:Tables are not properly aligned. Need to re look at the structure of the tables.

Response:

We appreciate this suggestion.

We have comprehensively revised the paper based on your comments and highlighted all the changes in the manuscript. The tables have been restructured.

Please see the attached revised manuscript.

We believe these revisions have significantly improved the manuscript, and we hope that it now meets the high standards of Plos One. Thank you once again for your valuable feedback.

Sincerely,

Shichuan Li

2024.7.18
